# *N*-glycosylation site occupancy in human prostaglandin H synthases expressed in *Pichia pastoris*

**DOI:** 10.1186/2193-1801-3-436

**Published:** 2014-08-15

**Authors:** Kaia Kukk, Sergo Kasvandik, Nigulas Samel

**Affiliations:** Department of Chemistry, Tallinn University of Technology, Akadeemia tee 15, 12618 Tallinn, Estonia; Proteomics Core Facility, Institute of Technology, University of Tartu, Nooruse 1, 50411 Tartu, Estonia

**Keywords:** Recombinant prostaglandin H synthase, PGHS, Cyclooxygenase, COX, *N*-glycosylation, Mass spectrometry, *Pichia pastoris*

## Abstract

Prostaglandin H synthases (PGHSs) are *N*-glycosylated membrane proteins that catalyse the committed step in prostaglandin synthesis. Unlike PGHS-2, the production of recombinant PGHS-1 in non-mammalian expression systems is complicated. The majority of the heterologous enzyme is inactive due to misfolding. Correct *N*-glycosylation is proposed to be obligatory for proper folding of mammalian PGHSs. In this study, human PGHS-1 and -2 (hPGHS-1 and -2) were expressed in the yeast *Pichia pastoris*. Recombinant hPGHS-2 was catalytically active, whereas hPGHS-1 was inactive. Accumulation of non-glycosylated hPGHSs was not observed in the crude lysate of the yeast cells. The *N*-glycosylation patterns of the purified recombinant proteins were characterised using nano-LC/MS/MS. The isoforms exhibited similar *N*-glycosylation site occupancy. The results indicate that there are more complex grounds for the inactivity of the recombinant hPGHS-1 produced in yeast.

**Electronic supplementary material:**

The online version of this article (doi:10.1186/2193-1801-3-436) contains supplementary material, which is available to authorized users.

## Background

**Figure 1:**
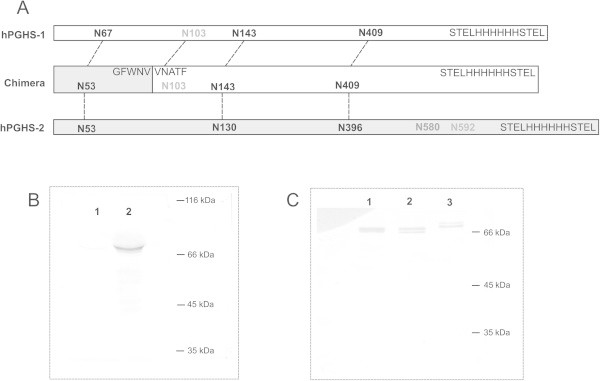
**Construction,** ***N-*** **glycosylation and expression of hPGHSs. A**, the positions of the *N*-glycosylation sites of the chimera and hPGHS isoforms. The dashed lines connect *N-*glycosylation sites that are conserved between isoforms. Asparagines, which are not glycosylated in native hPGHSs, are depicted with grey characters. N580 is designated by dark grey characters as about 50% of the native PGHS-2 is not glycosylated at this site. The transition from hPGHS-2 to hPGHS-1 to form the chimera is shown as well. **B**, Western blot analyses of the microsomes of *P. pastoris* cells expressing the non-optimised hPGHS-1 (1) and the codon optimised hPGHS-1 (2). hPGHS-1 was expressed with the native signal sequence and the optimised sequence contained an affinity tag. **C**, Western blot analysis of the purified hPGHSs subjected to *N*-glycosylation analysis: hPGHS-1 (lane 1), chimera (lane 2) and hPGHS-2 (lane 3). The calculated molecular weights of the non-glycosylated proteins were 67.2, 66.3 and 68.8 kDa, respectively, assuming that the signal peptide was cleaved and taking into account the double STEL and polyhistidine tag.

Prostaglandin H synthases (PGHSs, also known as cyclooxygenases or prostaglandin-endoperoxide synthases) are dimeric membrane proteins that catalyse the *bis*-oxidation of arachidonic acid (AA) to prostaglandin G_2_ (PGG_2_) and the subsequent reduction of PGG_2_ to PGH_2_. The downstream prostanoids play basic housekeeping as well as several pathophysiological roles in the body. Non-steroidal anti-inflammatory drugs inhibit the *bis*-oxidation reaction of PGHSs, thus blocking the key step in prostanoid synthesis. Vertebrates have two distinct PGHS isoforms: PGHS-1 and -2 (Kulmacz et al. 2003; Rouzer and Marnett 2009; Simmons et al. 2004; Smith et al. 2000b). The majority of the studies associated with mammalian PGHSs have been conducted with native or recombinant ovine PGHS-1 and recombinant human or murine PGHS-2 (Mbonye et al. 2006; Musee and Marnett 2012; Nemeth et al. 2001; Vecchio and Malkowski 2011; Vecchio et al. 2012). Thus there is little experimental data on human PGHSs (hPGHSs), particularly concerning hPGHS-1. Although the amino acid sequences of PGHS-1 and PGHS-2 are about 60% identical, unlike PGHS-2, the production of recombinant PGHS-1 in insect cells is obstructed as most of the enzyme is inactive and likely misfolded due to deficient glycosylation (Kulmacz et al. 2003; Shimokawa and Smith 1992). There are four and five *N*-glycosylation recognition sequons (N-X-S/T, X ≠ P) in the primary structures of hPGHS-1 and hPGHS-2, respectively (Figure 1A). N67, N143 and N409 (hPGHS-1 numbering) have been shown to be occupied in both isoforms and an additional site, N580 (human PGHS-2 numbering), in hPGHS-2 (Otto et al. 1993). The results of several studies indicate that *N*-glycosylation of these sites (except N580) is necessary for correct protein folding and/or oligomerisation (Otto et al. 1993; O’Neill et al. 1994). The glycosylation sites of murine PGHS-2 expressed in insect cells have been characterised showing that N53 and N130 (corresponding to N67 and N143 in hPGHS-1) are close to 100% glycosylated and that N396 (N409 in hPGHS-1) and N580 are partially glycosylated (Nemeth et al. 2001). hPGHSs contain 5 disulphide bonds, three of which are located in the epidermal growth factor (EGF)-like domain, whereas there is an 8 amino acid insertion in the N-terminal part of hPGHS-1 preceding the disulphide rich region (Kulmacz et al. 2003; Simmons et al. 2004). An ovine PGHS-1 mutant that had a disrupted disulphide bond (C59-C69) lacked activity, thus affirming the importance of an intact EGF-like domain for proper folding (Smith et al. 2000b).

The *N*-glycosylation pattern is highly variable among non-mammalian PGHSs. The primary structures of the red algal PGHSs from *Gracilaria vermiculophylla* (*G. vermiculophylla*) and *Coccotylus truncatus* contain two and three, the coral PGHS-A and PGHS-B from *Gersemia fruticosa* four and six, the coral PGHS from *Plexaura homomalla* three and the amphipod PGHSs from *Gammarus* and *Caprella* four and three *N*-glycosylation sequons, respectively (Järving et al. 2004; Valmsen et al. 2001; Varvas et al. 2009, 2013). Unlike animal PGHSs, the *G. vermiculophylla* PGHS does not require *N*-glycosylation for proper folding and expresses easily as a fully functional enzyme in *Escherichia coli* (*E. coli*) (Varvas et al. 2013). However, the expression of functional mammalian PGHSs requires the employment of a eukaryotic expression system. The yeast *Pichia pastoris* (*P. pastoris*) is capable of performing eukaryotic posttranslational modifications and therefore can be exploited for the production of recombinant mammalian glycoproteins (Macauley-Patrick et al. 2005). Recently, we used *P. pastoris* for heterologous expression of catalytically active hPGHS-2 (Kukk et al. 2012). hPGHS-1 expressed in similar conditions was inactive and solubilised poorly (personal data). The insufficient *N*-glycosylation of PGHS-1 in insect cells (Kulmacz et al. 2003; Shimokawa and Smith 1992) has led to the need for the characterisation of the *N*-glycosylation sites of hPGHS-1 produced in *P. pastoris*.

Mass-spectrometry based proteomics has become a standard method for the characterisation of post-translational modifications, including protein *N*-glycosylation (Pan et al. 2011). One way to map protein *N*-sites is to use a highly specific enzyme, PNGase F, which selectively hydrolyses *N*-oligosaccharides from respective peptides, thereby introducing specific mass-shifts. These shifts can be readily detected and localized with modern high resolution instruments. However, the reliability of such mapping is impaired by spontaneous deamidation that occurs in proteins and peptides under common alkaline sample preparation conditions. Thus artificial deamidation must be accounted for. An improved protocol was recently introduced using acidic pH with heavy water for the sample preparation of *N*-glycosylated proteins (Hao et al. 2011).

The aim of the present study was to investigate the *N*-glycosylation patterns of hPGHS-1 and hPGHS-2 produced in *P. pastoris* and verify whether insufficient *N*-glycosylation causes the misfolding of recombinant hPGHS-1. Therefore, hPGHS-1, hPGHS-2 and a chimera consisting of the two isoforms were expressed in *P. pastoris* and purified using Ni-affinity chromatography. The *N*-glycosylation site occupancy was characterised using nano-LC/MS/MS based proteomics. The structural characterisation of the oligosaccharides attached was not the primary objective.

## Results and discussion

### Expression of the recombinant hPGHSs in *P. pastoris*GS115

So far, functional mammalian PGHSs have been expressed in the baculoviral system (Vecchio and Malkowski 2011; Zou et al. 2012) and in mammalian cell lines (Mbonye et al. 2006, 2008), and hPGHS-2 recently in the yeast *P. pastoris* (Kukk et al. 2012). Here, the heterologous expression of hPGHS-2 was carried out as described by our group previously (Kukk et al. 2012), whereas similar expression conditions were employed for all the proteins subjected to study. The hPGHS-2 sequence contained a C-terminal polyhistidine tag and the native signal sequence was replaced with *P. pastoris* acid phosphatase secretion signal. Despite having a secretion signal, the protein was expressed intracellularly. Our previous study demonstrated that hPGHS-2 is produced in the yeast as a functional protein independent of the signal sequence (native or yeast) used (Kukk et al. 2012).

According to published data, in order to produce equal amounts of recombinant PGHS isoforms in the baculoviral expression system the heterologous expression of PGHS-1 requires extended expression time and scaling up of the insect cell culture volume approximately three times (Smith et al. 2000a). Similarly, the production of hPGHS-1 in *P. pastoris* proved complicated. At first, we subjected native hPGHS-1 to heterologous expression; however, the expression level of the protein was very low (Figure 1B, lane 1). According to the Graphical Codon Usage Analyser (http://gcua.schoedl.de), the codon usage of hPGHS-1 is less compatible with the yeast *P. pastoris* than that of hPGHS-2. Therefore, the codon usage was adjusted. In addition, GC content was optimised, potential mRNA instability elements were removed and yeast Kozak consensus sequence (AAAA**ATG**TC) was used to initiate translation. The sequence encoding a polyhistidine tag was inserted into the C-terminal end, and the four amino acid residues preceding the tag (STEL) were repeated after the tag (Figure 1A). The native sequence of hPGHS-1 contained an unwanted *Eco*R I restriction site that was removed. The synthetic sequence has been submitted to GenBank with the accession number KM112253. The Western blot analysis of the microsomes of the recombinant *P. pastoris* cells revealed that the expression level of the codon optimised hPGHS-1 was remarkably higher (Figure 1B, lane 2). Nevertheless, the crude cell preparation did not exhibit detectable cyclooxygenase activity (Table 1). In addition, the poor solubilisation of hPGHS-1 indicated that the protein was mostly misfolded.

**Table 1:** **hPGHSs subjected to** ***N*** **-glycosylation analysis**

Recombinant vector	Native/ optimised sequence	Signal sequence	Activity ^1^
optC-termHis_6_PGHS-1 pHIL-D2	Optimised	Native	0
N-termPGHS-2/optC-termHis_6_ PGHS-1 pHIL-D2	Optimised, except the sequence of PGHS-2	PGHS-2	215 ± 19
C-termHis_6_PGHS-2-sp pHIL-S1	Native	Yeast	577 ± 21

^1^pmoles of prostaglandins formed in 10 min per 1 mg (wet weight) of yeast cells. Mean ± S.D. (n = 5).

Conservation of the amino acid sequence between PGHS-1 and -2 in the membrane binding domain (MBD) is about half of overall protein identity (33% versus 60%). In addition, the length of the N-terminal signal peptide is remarkably different – 17 and 23 amino acids in PGHS-1 and -2, respectively (Kulmacz et al. 2003). As hPGHS-1 expressed as an inactive protein, the region of hPGHS-1 that has low amino acid sequence similarity with hPGHS-2 was replaced with the respective sequence of hPGHS-2. More precisely, an N-termPGHS-2/optC-termHis6 PGHS-1 chimera was created that consisted of the N-terminal part of hPGHS-2, including the first three helices of MBD and the C-terminus of hPGHS-1 (Figure 1A), whereas the hPGHS-1 sequence was optimised for the yeast. The fourth helix of MBD merges into the catalytic domain (Smith et al. 2000b) and was, therefore, left unaltered. The recombinant chimeric protein exhibited better solubilisation characteristics compared to hPGHS-1 and exhibited detectable catalytic activity. The characteristics of the hPGHSs subjected to *N*-glycosylation analysis are presented in Table 1.

### *N*-glycosylation analyses

**Figure 2:**
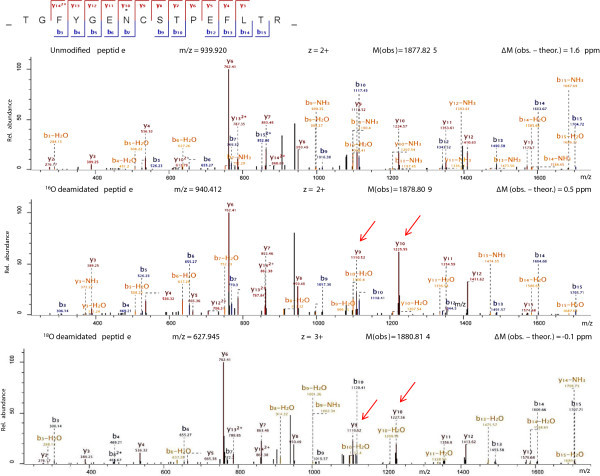
**Example fragmentation spectra of a PGHS-1** ***N*** **-glycosylation site containing peptide TGFYGENCSTPEFLTR, indicating variable glycosylation.** High mass accuracy of precursor peptides combined with MS/MS fragment ions readily captures expected ^16^O/^18^O dependent mass-shifts and confidently identifies that the site has variable glycosylation state in the recombinant protein. y-series fragment ions which pinpoint the modification within the sequence are indicated with red arrows (note, that beyond y9-fragment the masses are shifted relative to the unmodified fragments according to the incorporated oxygen atom).

The results of the analysis are summarised in Table 2. Depending whether the asparagine residues were unmodified or deamidated by H_2_^16^O or H_2_^18^O it was possible to discriminate between non-glycosylated (unmodified and ^16^O deamidated) and glycosylated (^18^O deamidated) sites. Example MS/MS spectra are presented in Figure 2 of a glycosylation motif containing peptide in which variable site occupancy can be confidently deduced. Using the described protocol we observed 79 MS/MS events regarding ^18^O deamidated peptides of PGHS proteins. All were found to contain *N*-glycosylation sequons and 70 scans also precisely localised the modification to the expected residue. Only one peptide (YNYQQFIYNNSILLEHGITQFVESFTR; PGHS-2) had 3 out of 15 MS/MS scan events where the ^18^O modification was not on an *N*-sequon asparagine, 6 were localized to the expected residue and 6 were ambiguous between the two consecutive asparagines contained in the sequon. Therefore, we conclude that the *N*-mapping approach produced confident assignments with very low level of conflicting data. The complete list of the identified peptides is presented in Additional file 1: Table S1 and the respective mass spectrometry proteomics data have been deposited to the ProteomeXchange Consortium (http://proteomecentral.proteomexchange.org) (Vizcaino et al. 2013) via the PRIDE partner repository with the dataset identifier PXD000965.

**Table 2:** **Mass-spectrometric analysis of the** ***N*** **-glycosylation patterns of hPGHS proteins**

Protein	Enzyme(s) used	Glycosylation site	^18^O deamidation	^16^O deamidation	Unmodified ***N-***sequon	Glycosylation state
**hPGHS-1**	AspN + trypsin + GluC	N67	+	+	+	Variable^II-III^ / Yes^I^
		*N103* ^1^	-	+	+	No
		N143	+	-	+	Variable^II-III^ / Yes^I^
		N409	-	-	-	ND^2^
**hPGHS-2/-1 chimera**	AspN + trypsin	N53	+	+	+	No^II^ / Yes^I^
		*N103* ^1^	-	+	+	No
		N143	+	-	-	Yes
		N409	+	-	-	Yes
**hPGHS-2**	trypsin	N53	+	+	+	No^II-IV^ / Yes^I^
		N130	+	+	+	Variable^III-IV^ / Yes^I-II^
		N396	+	+	+	Variable^III-IV^ / Yes^I-II^
		N580	+	-	-	Yes
		*N592* ^1^	-	-	+	No

^1^ *N*-glycosylation sequons not glycosylated in native hPGHS proteins.

^2^no data.

^I-IV^hPGHS glycoforms, whereas I marks the protein with the highest molecular weight. If not depicted, the glycosylation state of the sequon was the same among all the glycoforms subjected to analysis.

The analysis of the two dominant glycoforms of hPGHS-2 (Figure 1C, lane 3) revealed that alternative glycosylation at N53 differentiated the glycoforms. Both glycosylated and non-glycosylated peptides were observed for N130 and N396 indicating variable *N*-glycosylation of the sites in lower molecular weight glycoforms. The fourth glycosylation sequon at N580 was glycosylated. Therefore, *N*-glycosylation of PGHS-2 in yeast and insect cells (Nemeth et al. 2001) differ from each other.

The two electrophoretic bands of N-termPGHS-2/optC-termHis_6_ PGHS-1 chimera (Figure 1C, lane 2) were analysed separately. In accordance with the site occupancy of hPGHS-2, the double band turned out to be the result of alternative glycosylation at N53 (hPGHS-2 numbering). Although N103 constitutes the *N*-glycosylation sequon the site was not occupied in the recombinant chimera produced in *P. pastoris*. The next two *N*-glycosylation sites were occupied. Thus approximately 50% of the protein was properly glycosylated. It may be speculated that the extensive rearrangement of the amino acid sequence of hPGHS-1 to form the chimera caused the low protein activity.

The majority of hPGHS-1 remained in the microsome fraction and was excluded from subsequent analysis. According to Western blot analyses of the solubilised protein fraction and the remaining microsome, both hPGHS isoforms exhibited similar protein patterns. Specifically, both fractions contained multiple glycoforms with a slight preference for the less *N*-glycosylated proteins for the microsome. In addition, there were no noticeable protein bands corresponding to non-glycosylated hPGHSs, as was expected in terms of insufficient *N*-glycosylation. The purified hPGHS-1 appeared as a double electrophoretic band, whereas the molecular weight difference between the glycoforms was less noticeable than in the case of hPGHS-2 and the chimera (Figure 1C). The peptides containing the first three sequons were identified, whereas N103 was not glycosylated and N67 and N143 were variably glycosylated. Taking into account the glycosylation of hPGHS-2 and the chimera, alternative glycosylation at N67 probably differentiated the dominant glycoforms. The fourth site was not identified. Nevertheless, the analysis of the chimera clearly confirmed that the sequon was glycosylated. In addition, the electrophoretic mobility of the glycoforms of hPGHS-1 and the chimera (Figure 1C, lanes 1 and 2) indicated that three sequons were occupied in the highest molecular weight protein. Specifically, the protein band of the chimera with three oligosaccharides appeared lower than the putative respective glycoform of hPGHS-1, whereas the calculated molecular weights of the non-glycosylated chimera and hPGHS-1 were 66.3 and 67.2 kDa, respectively. Therefore, the *N*-glycosylation pattern of the purified hPGHS isoforms was similar.

Unlike hPGHS-2, the purified hPGHS-1 was catalytically inactive. The crude lysate of the yeast cells expressing the chimera exhibited detectable cyclooxygenase activity, which may indicate that the cause of the inactivity of hPGHS-1 lies in the N-terminal region of the protein. The PGHS-1 specific 8 amino acid N-terminal insertion was removed in the chimera. It may be that the insertion has a detrimental effect on the disulphide formation in yeast. Incomplete disulphide formation in *P. pastoris* has been described for *Stereum purpureum* endopolygalacturonase I (Ogawa et al. 2009) and human consensus interferon-alpha mutant (Wu et al. 2010). Unfortunately, the positions of the disulphide bonds of hPGHSs could not be determined simply by mass spectrometry, as the cysteines involved in bonding are very close together in the sequence. The known pattern of disulphides of mammalian PGHSs has been established from the crystal structures (Kulmacz et al. 2003). Partial reduction of disulphide bonds has been described for the analysis of disulphide rich proteins (Foley et al. 2008). However, optimising the conditions for the partial reduction and alkylation requires significant amounts of recombinant protein, which becomes an obstacle when studying a relatively challenging membrane protein.

The less noticeable molecular weight difference between the dominant glycoforms of hPGHS-1 may result from a shorter oligosaccharide. The transfer of completely assembled oligosaccharide to protein is favoured (Aebi 2013). In yeast, however, if the complete oligosaccharide is not available, incompletely assembled oligosaccharides are transferred (Helenius and Aebi 2004). Under the conditions where the yeast cells are forced to over-express an aggregation-prone protein there may be a lack of enzymes performing the biosynthesis of lipid-linked oligosaccharides. The increased stress is also reflected in lower cell densities for cultures expressing the misfolded recombinant protein.

The partial occupancy of the first *N*-glycosylation site of hPGHSs was rather surprising. The sequon might not have been fully glycosylated because the cysteine at position +1 was involved in disulphide bonding, and disulphide bond formation can compete with *N*-glycosylation (Allen et al. 1995; Daly and Hearn 2005). Over-expression of *Leishmania major* STT3D, a subunit of the oligosaccharyltransferase (OST), under the control of an inducible alcohol oxidase 1 promoter, improved the *N-*glycosylation site occupancy of the recombinant antibodies to greater than 99% (Choi et al. 2012). Similar co-expression with hPGHSs might increase the proportion of the fully *N*-glycosylated protein.

The non-occupancy of the *N-*glycosylation sequon at N103 in hPGHS-1 has barely been discussed in relevant papers. It has been reported that the replacement of hydrophobic amino acids in membrane binding helices B and C with smaller neutral or hydrophilic residues results in *N*-glycosylation at N103 (Spencer et al. 1999), and the N103Q mutant of ovine PGHS-1 retains 45-50% of both peroxidase and cyclooxygenase activities (Otto et al. 1993). The analysis of the protein environment of *N*-glycosylation sites revealed that the lowest incidence of the occupied sequons was on helices and the highest on flexible loops at or after points where the secondary structure changes (Petrescu et al. 2004; Zielinska et al. 2012). The distance from a transmembrane domain may also influence *N*-glycosylation (Nilsson and von Heijne 1993). In agreement with the data reported previously on ovine PGHS-1 (Otto et al. 1993), our study demonstrates that the sequon at N103 in recombinant hPGHS-1 is not occupied. Although PGHS-1 does not contain transmembrane helices but is rather a monotopic membrane protein (Picot et al. 1994), the location of the sequon at the C-terminal end of helix C in MBD may hinder the *N*-glycosylation process of that sequon.

To date it is not established clearly why PGHS-1 is produced predominantly in a misfolded state in non-mammalian expression systems. The beginning of the *N-*glycosylation process is similar in all eukaryotes. In the mammalian Golgi apparatus, a series of trimming and addition reactions are performed that generate high-mannose (five to six mannose residues), and complex or hybrid oligosaccharides. The secreted proteins of *P. pastoris* typically have oligosaccharide chains that contain eight to nine mannose residues (Cereghino and Cregg 2000; Montesino et al. 1998). It has been proposed that the longer chains may interfere with the folding or functionality of a heterologous mammalian protein (Montesino et al. 1998). OST is a heterooligomeric complex that plays a central role in protein *N*-glycosylation. Mammalian OST contains components that are homologous to the yeast OST. Nevertheless, there are components unique to the mammalian complex (Aebi 2013; Helenius and Aebi 2004; Mohorko et al. 2011). Therefore, it may be speculated that the yeast OST complex is sufficient for *N*-glycosylation of PGHS-2, whereas proper folding of PGHS-1 requires specific mammalian OST subunits not found in yeast. However, the data obtained in our study indicate that the *N*-glycosylation site occupancy of the PGHS isoforms is rather similar. It is tempting to speculate that the exact composition or length of the oligosaccharide is critical for proper folding of hPGHS-1. Insufficient or improper disulphide formation also cannot be ruled out.

## Methods

### Expression and purification of hPGHSs

The codon-optimised cDNA of hPGHS-1 (GenBank accession number KM112253) in the pMK-RQ-Bb vector (GeneArt, Life Technologies) was amplified using *E. coli* DH5α. The sequence encoding hPGHS-1 was ligated into the yeast vector pHIL-D2 (Life Technologies) using the *Eco*R I restriction site. N-termPGHS-2/optC-termHis_6_ PGHS-1 fusion was achieved as follows: the N-terminal part of hPGHS-2 (OriGene), including the first three helices of the MBD, was amplified using the primers hC2(*Eco*R I)up (5’ TTCGAATTCCGGATGCTCGCCCGCGCCCTGCTGC 3’) and hC2(*Hpa* I)down (5’ GAATGTTGTTAACAACGTTCCAAAATCC 3’); the C-terminal part of the codon-optimised hPGHS-1, starting with helix D, was amplified using opthC1(*Hpa* I)up (5’ GGGAGTTCGTTAACGCTACTTTCATCAG 3’) and opthC1 C-termHis_6_(*Eco*R I)down (5’ GCCGAATTCTTACAACTCAGTGGAGTGG 3’) (DNA Technology). The PCR products were double digested with *Hpa* I and *Eco*R I (Thermo Scientific) and ligated into the *Eco*R I linearised pHIL-D2 vector. The recombinant vector was amplified using *E. coli* DH5α and TOP10F’.

The correctness of the sequences was confirmed by DNA sequencing (LGC). The recombinant expression vectors were linearised with *Pme* I (Thermo Scientific) and transformed into the *P. pastoris* strain GS115 (Life Technologies) using the spheroplast method (Cregg et al. 1985). The presence of the hPGHS-1 encoding sequences was confirmed by direct PCR of the *P. pastoris* colonies, using the following upstream and downstream primers: optQEV-up (5′ CCAGATGGCTGTTGGTCAAGAGG 3′) and optTWG-down (5′ GGAACAACTGTTCATCACCCCAAG 3′). Construction of the pHIL-S1 expression vector encoding hPGHS-2 has been described elsewhere (Kukk et al. 2012). The heterologous expression and protein purification were carried out as described previously (Kukk et al. 2012). Briefly, methanol induction was carried out at 20°C for 72 hours, the yeast cells were disrupted by sonication and the detergent-solubilised hPGHSs were purified using Ni-affinity chromatography.

### SDS-PAGE and Western blot analysis

Microsomes were prepared as reported previously (Kukk et al. 2012). A 10% SDS-polyacrylamide gel with a 4% stacking gel was used to separate the microsomal or purified protein sample. The proteins were transferred from the polyacrylamide gel onto the Protran BA85 nitrocellulose membrane (Whatman) using a Trans-Blot Semi-Dry apparatus (Bio-Rad) and the Bjerrum and Schafer-Nielsen transfer buffer (48 mM Tris, 39 mM glycine, 1.3 mM SDS and 20% methanol). Mouse PGHS-1 specific (Life Technologies) or mouse PGHS-2 specific monoclonal antibody (BD Biosciences) was used as the primary antibody. Alkaline phosphatase conjugated goat anti-mouse IgG (LabAs, Estonia) was used as the secondary antibody. The bands were visualized with nitro blue tetrazolium and 5-bromo-4-chloro-3-indolyl phosphate (Sigma-Aldrich).

### Detection of the hPGHS activity by incubation with ^14^C-labeled arachidonic acid

The activity of hPGHSs was detected as described previously (Kukk et al. 2012). Briefly, the enzyme preparation was incubated with 50 μM [1-^14^C] AA (500 cpm/μl) (GE Healthcare) with intensive stirring for 10 min and the products were extracted with ethyl acetate and analysed by thin layer chromatography using authentic reference standards of prostaglandins D_2_, E_2_ and F_2α_. A Wallac 1410 Liquid scintillation counter was used to count the ^14^C label in stains. The extent of conversion of AA into prostaglandins was calculated and expressed as the hPGHS activity in picomoles of prostaglandins formed in 10 min per 1 mg (wet weight) of yeast cells.

### In-gel digestion and deglycosylation of hPGHSs

The purified protein sample was separated in 7% SDS-polyacrylamide gel. The gel was stained with Coomassie Brilliant Blue R-250 and destained with 40% methanol and 10% acetic acid (AcOH). The region containing the protein of interest (0.5-1 μg) was cut from the gel and sliced into approximately 1 mm^3^ pieces. In-gel digestion and subsequent deglycosylation were carried out using a slightly modified method described by Hao et al. ( 2011). Briefly, the gel samples were destained by vortexing in 1:1 acetonitrile (ACN): 50 mM ammonium acetate (AmAc), pH 6 for 30 min. Then, the samples were reduced with 10 mM dithiothreitol at 56°C and alkylated with 50 mM iodoacetamide for 20 min in the dark. The gel pieces were dehydrated with ACN and dried under a hood. In-gel digestion was carried out for 2 h on ice and then overnight at 37°C with 50 μl of 10 ng/μl proteomics grade trypsin (Sigma), or a cocktail of sequencing grade Asp-N (Promega), Glu-C (Promega) and trypsin in 100 mM ammonium bicarbonate buffer, pH 7.8. Peptides were extracted from the gel by sonication for 5 min, followed by vortexing in 2 volumes of 1:2 5% formic acid (FA): ACN for 30 min. The solution was dried in a vacuum-centrifuge and the peptides were reconstituted to 40 μl with 200 mM AmAc, pH 5 buffer in 98% H_2_^18^O (Sigma). Then, 1 μl of PNGase F (New England Biolabs) was added and the deglycosylation mix was incubated at 37°C for 12 h. Finally, the peptides were purified on C18 StageTip (Rappsilber et al. 2003) and reconstituted with 0.5% trifluoroacetic acid.

### Nano-LC/MS/MS analysis of deglycosylated hPGHS peptides

Peptides were separated on an Agilent 1200 series nano-LC with in-house packed (3 μm ReproSil-Pur C18AQ particles) 15 cm × 75 μm ID emitter-columns (New Objective) using an 8-50% gradient of buffer B for 1 h, whereas buffer A was 0.5% AcOH in water and B 0.5% AcOH in 80% ACN. Separated peptides were eluted at 200 nl/min (spray voltage 2.0-2.2 kV) to a LTQ Orbitrap XL (Thermo Fisher Scientific) mass-spectrometer operating with a top-5 MS/MS strategy with a minimum of 1 s cycle time. The maximum ion injection times were 500 ms, the dynamic exclusion was set to 60 s and only charge states over +1 were analysed. Alternatively, the peptides were separated on an UltiMate 3000 RSLCnano (Dionex) using a cartridge trap-column in backflush configuration and an analytical 50 cm Easy-spray column (75 μm ID, 2 μm C18 particles) operated at 40°C and 1.8-2.1 kV. Peptides were eluted at 200 nl/min using the previously mentioned gradient (except that 0.1% FA was used instead of AcOH) to a Q Exactive MS/MS (Thermo Fisher Scientific) operating with a top-10 strategy with a maximum cycle time of 1 s (dynamic exclusion set to 20 s).

### Mass-spectrometric data analysis

Raw data were processed with the MaxQuant 1.4.0.8 software package (Cox and Mann 2008). Spectra were searched against UniProt (http://www.uniprot.org) *P. pastoris* complete proteome database (2013 September) supplemented with PGHS sequences and common contaminants. Missed cleavages were set to 2 and only identifications with a minimum of 2 peptides 6 amino acids long were accepted. The protein and peptide false discovery rate was set below 1%. Methionine oxidation, asparagine and glutamine deamidation (with and without ^18^O) were set as variable modifications. Carbamidomethylated cysteines were set as fixed modifications. It was noted that in some samples C-terminal oxygens were also extensively replaced with ^18^O, indicating residual trypsin activity in the deglycosylation mix. For those cases, analysis was redone using C-terminal ^18^O double substitution as an additional fixed modification. All other parameters were default.

## Electronic supplementary material

Additional file 1: Table S1: Complete list of the identified peptides of PGHS proteins. (XLSX 434 KB)

## Abbreviations

AA: Arachidonic acid
ACN: Acetonitrile
AcOH: Acetic acid
AmAc: Ammonium acetate
*E. coli*: *Escherichia coli*
FA: Formic acid
*G. vermiculophylla*: *Gracilaria vermiculophylla*
h: Human
MBD: Membrane binding domain
OST: Oligosaccharyltransferase
*P. pastoris*: *Pichia pastoris*
PGG_2_: Prostaglandin G_2_
PGHS: Prostaglandin H synthase.
